# Gender and Age Differences in Loneliness: Evidence for People without and with Disabilities

**DOI:** 10.3390/ijerph17249176

**Published:** 2020-12-08

**Authors:** Ricardo Pagan

**Affiliations:** Department of Applied Economics, University of Malaga, 29071 Malaga, Spain; rpr@uma.es

**Keywords:** loneliness, disability, gender inequality, age

## Abstract

This study examines the relationships between loneliness, gender, and age for people without and with disabilities (moderate versus severe) in Germany. Using data taken from the German Socio-Economic Panel (SOEP) for the years 2013 and 2017 and using the UCLA (University of California, Los Angeles) Loneliness Scale, in general we found that males report lower loneliness scores as compared to those for females. Furthermore, we found a strong association between loneliness and the individual’s age, but with differences according to gender and disability status. For example, for males with severe disabilities levels of loneliness decrease with age, whereas for females with severe disabilities the opposite result is found. In addition, we found that participation in leisure activities and having a higher frequency of contacts with family, friends, and social online networks (measured by the relational time index) contribute to reducing loneliness for all individuals. From a public policy perspective, it is necessary to undertake the design, promotion, and implementation of instrumental, emotional, and social support measures for people with disabilities (in particular for females that are severely limited in their daily activities), which can contribute to reducing their loneliness scores and increasing their levels of life satisfaction.

## 1. Introduction

The deficit in social participation and relationships can lead individuals to report higher levels of loneliness [1,2,3,4]. Social relationships are also crucial to personal health and happiness [5,6,7,8,9]. Loneliness can be defined as the discrepancy between desired and actual quantity and quality of social relations [10]. Furthermore, loneliness is a subjective experience, which is painful, unwanted, aversive, and difficult to tolerate [11,12,13]. It has also been associated with alcohol abuse and suicide, and undermines confidence in the ability to create and maintain social relationships [14,15,16,17,18,19]. Loneliness scores vary by personality, age, gender, regions, cultures, race, socio-economic conditions, and health status, among other factors [20,21,22,23,24,25,26].

According to the literature on loneliness and health status [27,28,29,30,31,32,33,34], loneliness is a strong predictor of mental health problems, depression, heart disease, blood pressure issues, poorer health status, and functional decline, and is a risk factor for mortality and morbidity. In many cases, and in particular among older people, these illnesses and health concerns may lead individuals to experience functional disabilities, thus reducing their levels of social contact, engagement with other peers, and participation in leisure activities [35,36,37,38,39]. People with disabilities are more vulnerable to discrimination, stigmatization, marginalization, and social exclusion, and hence more likely to report higher levels of loneliness than people without disabilities [40,41,42]. The loss of independence, autonomy, self-esteem, and confidence among people with disabilities may seriously affect their loneliness scores. Furthermore, disability rates increase with age and are traditionally higher for females than males, while females also have a longer duration of life lived with a disability [43].

The goal of this study is to analyze the levels of loneliness reported by people without and with disabilities by gender and age in Germany. We use data taken from the German Socio-Economic Panel (SOEP) for the years 2013 and 2017. The use of this dataset allows us to divide people with disabilities into two groups: those people who have a moderate disability, and those with a severe disability that limits their daily activities. In addition, for those two years, the SOEP includes a set of questions that are suitable for creating a validated three-item version of the UCLA Loneliness Scale [44,45,46]. Although there is a plethora of previous studies investigating loneliness, few empirical studies exist that analyze the association between loneliness and disability [38,39,40,41,47,48]. However, none of these studies have examined the loneliness differential by gender and age for people with disabilities. Looking at the existing literature on loneliness, we also found inconsistent results in terms of gender and age differences in loneliness for the general population. For example, several studies [36,49,50] have found that males are more likely than females to report lower levels of loneliness, whereas others found the opposite result [51,52]. In the same vein, the relationship between age and loneliness is unclear. For example, Yang and Victor [22] found that the prevalence of loneliness generally increases with age, but this relationship varies by country, with those individuals living in Northern European countries reporting lower levels of loneliness across the age groups than those in Southern Europe. In contrast, other studies have found a U-shaped relationship between loneliness and age, in which younger and older people report higher levels of loneliness as compared to those in middle age [23,53,54].

Overall, none of these previous empirical studies have analyzed the particular situation of people with moderate and severe disabilities in terms of loneliness in general, and gender and age differences in particular. To our knowledge, this is the first attempt to investigate these issues and shed further light on predictors of loneliness at different development stages of life for males and females with disabilities. Therefore, this study fills an important gap in the existing literature on loneliness and disability, and our results may help design and implement specific public actions and policies to increase the levels of social participation, inclusion, and well-being among people with disabilities.

## 2. Data and Methods

To carry out this study, we used data taken from the German Socio-Economic Panel (SOEP) for the years 2013 and 2017. This survey was developed and is conducted by the German Institute for Economic Research (DIW Berlin), and funded by the German Federal Government and the State of Berlin. This representative longitudinal dataset of German individuals started in 1984 and has a high level of stability over time (thanks to the use of refreshment samples in 1998, 2000, 2006, 2011, 2012, and 2017). One of the main advantages of the SOEP is the rich and varied information it contains at individual and household levels on income, living conditions, employment, health status, household composition, education, social capital, and satisfaction, among others (for example, see Wagner et al. [55], Goebel et al. [56], and https://www.diw.de/en/soep for additional information on the SOEP data, samples, consent from the respondents, questionnaires, and methodology, among others). Despite the fact that the SOEP dataset has information for the period 1984–2018 (version 35), we only used data from two years (2013 and 2017), because the loneliness measure was only available within those years. In our case, we restricted our sample to individuals aged 16 or over and without missing information. After dropping observations with missing values, our final samples used for the loneliness regressions consisted of 19,763 and 22,806 males and females, respectively.

Similar to other previous studies on loneliness [23,45,57,58], we constructed our loneliness measure following the work of Russell [44], a validated three-item version of the UCLA Loneliness Scale, designed specifically for large datasets. For this purpose, we used three questions included in the SOEP for the years 2013 and 2017:

(1) How often do you miss the company of other people?
(2) How often do you feel left out?
(3) How often do you feel socially isolated?

The possible answers to these three questions and the codification used in this study were: 0 = never; 1 = seldom; 2 = sometimes; 3 = often; 4 = very often. In line with other studies [23,59,60], we constructed our loneliness measure (called “loneliness”) as the mean value of the responses to these three questions, i.e., the loneliness scale takes values from zero (no loneliness) to 4 (high level of loneliness). According to Hughes et al. [45], this three-item version of the UCLA Loneliness Scale is strongly correlated with the full 20-item version of the UCLA Loneliness Scale (and also with depression and perceived stress). In addition, Hawkley et al. [46] found that the German version used in the SOEP exhibits invariance and to correlate similarly with correlates of loneliness, such as self-rated health and frequency of social activity.

As for our disability measure, once again we used two questions from the SOEP for the years 2013 and 2017:

(1) Do you have a health problem that limits you in normal everyday life? Possible answers: Yes, severely; yes, somewhat; no, not at all;
(2) For those individuals responding “yes, severely” or “yes, somewhat” to the first question, the follow up question was: Have you had this health problem for more than half a year? Possible answers: Yes/no.

Those individuals who responded “no, not at all” to the first question were defined as “people without disabilities”. In addition, those individuals who responded “no” to the second question were again considered as “people without disabilities”, whereas those answering “yes” were defined as “people with disabilities”. Within this latter group and following the work of Gannon [61], we differentiated two subgroups of people with disabilities according to their degree of disability: (a) those reporting a health problem for more than half a year that severely limits their normal daily activities; (b) those reporting such a condition but state that it limits them somewhat (i.e., moderately). Therefore, we identified three possible groups by disability status:

(1) People without disabilities;
(2) People with disabilities, moderately limited;
(3) People with disabilities, severely limited.

We used a definition of disability that “is a standard measure used in many OECD countries – it conforms to the newer social model of disability, whereby disability is seen as a consequence of social, attitudinal, and environmental barriers that prevent people from participating in society (Gannon and Munley [62], p. 40)”. The European Disability Strategy 2010–2020 also adopted this new social model to design and implement actions and policies aimed at achieving full economic and social participation of people with disabilities across Europe. Although our disability measure is a self-evaluation and does not refer to an “objective” definition of disability, the questions of the SOEP incorporate the main objective of the World Health Organization definition, which relates disability to limitations on daily activities. In any case, we have to bear in mind that that measuring the degree of disability with any survey is inherently difficult from a methodological point of view and is subject to wide debate among the people involved [63].

As for the econometric process, we ran an ordinary least squares (OLS) model on loneliness, which was performed separately for males and females. We also ran this OLS model for different age groups in order to found any different effect of disability on loneliness. In particular, we split our sample into four age groups: 16–29 years (young people), 30–49 years (lower middle-aged people), 50–64 years (higher middle-aged people), and 64 years and above (older people). To estimate our OLS model of loneliness and following the existing literature, we included the following set of explanatory variables: individual’s disability status (i.e., without disabilities, moderate disability, or severe disability), age (and its square and cube values divided by 1000), years of education, marital status (i.e., single, living without a partner, or living with a partner), existence of children in the household, household size, having German nationality, real household income (in logarithms), employment status (i.e., full-time, part-time, or non-working), relational time index (proposed by Becchetti et al. [64], measuring the individual’s social capital, which is simply the unweighted mean (i.e., all the components have the same weight, thus allowing for perfect substitution between the relational events) of the scores given to the frequency of contacts with family members, friends, and neighbors; use of online social networks; frequency of participation in volunteering, sports, cultural activities, and religious events), region of residence (17 German states/Länder), and year of interview (i.e., 2013 or 2017). We used the statistical package STATA 16 to carry out all descriptive and econometric analyses. 

## 3. Results

**Figure 1:**
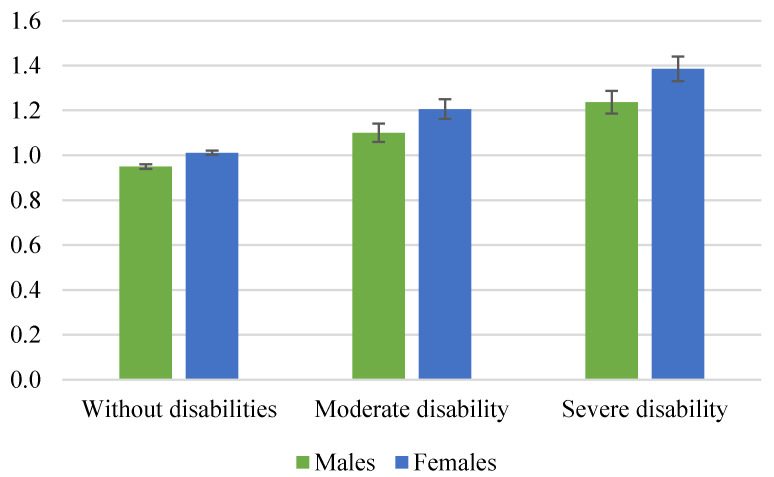
Mean loneliness scores by disability status for German males and females. Note: Weighted data. Individuals aged 16 or over. Confidence intervals (*p* < 0.05) are shown. Number of observations: 20,841 males (18,131 without disabilities, 1415 with a moderate disability, and 1295 with a severe disability); 26,445 females (23,800 without disabilities, 1425 with a moderate disability, and 1220 with a severe disability). Source: SOEP data for the years 2013 and 2017.

Figure 1 shows the mean loneliness scores for males and females by disability status in Germany. Overall, we found that females were more likely to report higher loneliness scores than males. For example, for males without disabilities, the mean loneliness score was 0.95 points, whereas for their female counterparts, this went up to 1.01 points (i.e., the loneliness differential was 0.06 points, which was statistically significant according to the confidence intervals (*p* < 0.05)). For people with moderate and severe disabilities, the gender differences in terms of loneliness were 0.106 and 0.149 points in favor of females, respectively (which again are statistically significant). Namely, the loneliness differential between males and females increases as the degree of disability severity increases. In this vein, it is worthwhile comparing the mean loneliness by disability status. Using the mean loneliness scores reported by “males without disabilities” as a reference, for males we detected that the loneliness differences were 0.151 and 0.286 points for males with moderate and severe disabilities, respectively (and statistically significant at a 95% level according to a test of means). For females, these loneliness differentials are even greater (and statistically significant) than those found for males, i.e., 0.195 and 0.375 points for females with a moderate and a severe disability, respectively. For people with disabilities (especially those with severe disabilities), the existence of low self-esteem, confidence, and social skills may contribute to limiting and inhibiting their social interactions, thus increasing their loneliness scores [47].

Looking at the existing literature on loneliness and gender, Pinquart and Sörensen [36] point out that females report greater levels of loneliness than males because they have higher risks for widowhood, living alone, chronic illness, disability, and functional limitations. Moreover, females tend to require more care in later life than males [65], and those aged 65 can live with a disability 6.9 years more than males [66]. Married females may also face a greater risk of loneliness if they take care of a spouse with a disability [41,67]. Finally, we also considered that females tend to admit and recognize their real levels of loneliness more easily than males [68]. In contrast, males have a lower social acceptance of loneliness (because of potential problems of stigmatization) than females, especially during adolescence and young adulthood [68].

**Figure 2:**
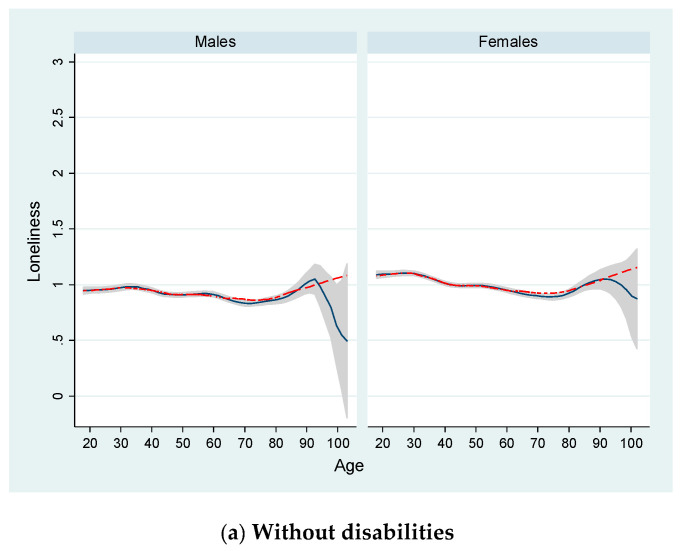

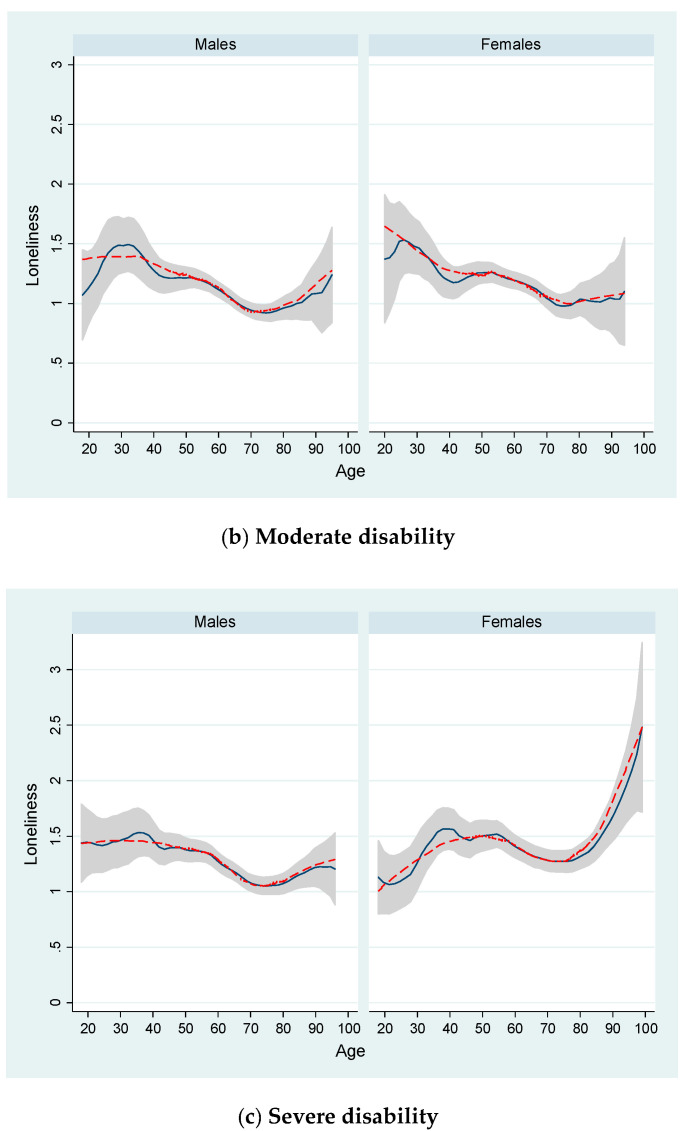
Mean loneliness distribution by age and disability status for German males and females. Note: Individuals aged 16 or over. LOWESS (red dashed lines) and LPOLY (black lines with confidence intervals (*p* < 0.05) in grey) smoothing functions shown. For the number of observations used to obtain these results, see the notes in Figure 1. Source: SOEP data for the years 2013 and 2017.

Figure 2 shows the age distribution of loneliness for males and females according to their disability status. For this purpose, and similar to Luhmann and Hawkley [23], we calculated “locally weighted scatterplot smoothing (LOWESS)” and “kernel-weighted local polynomial regression (LPOLY)” functions. These techniques allowed us to display a graph with smoothed values (and with confidence intervals when using LPOLY). For example, these smoothed LOWESS values are obtained by running a regression of yvar = “loneliness” on xvar = “age” by using only the data for (xi, yi) and a few other data points near this point. In LOWESS, the regression is weighted so that the central point (xi, yi) gets the highest weight and points that are farther away (based on the distance |xj − xi|) receive less weight [69]. In addition, LOWESS tends to follow the data (because of its locality), whereas LPOLY fits a local pth-order polynomial and is global, i.e., what happens on the extreme left of a scatterplot can affect the fitted values on the extreme [69].

For people without disabilities, we found very similar age distributions for males and females. Around the age of 30 and 35 for both females and males, we found a steady decrease in the levels of loneliness until around the age of 74. From this age onwards for both males and females, loneliness increases continuously (according to LOWESS) or reaches a peak around the age of 95 (according to LPOLY), although the size of the confidence interval is larger due to the low number of observations at those ages. Looking at the age distribution for people with a moderate disability, we found a well-defined and non-linear loneliness pattern, with a peak around the ages of 26 and 32 for females and males, respectively (according to LOWESS). There was a clear downward trend from these ages until around the ages of 70 (males) and 74 (females), which then increased uninterruptedly from this point (although at a much lower rate among females). As for people with a severe disability, we detected significant age differences in terms of loneliness between males and females. For males and according to LOWESS, we found a steady trend from the youngest ages to middle adulthood (around age 55), following a negative trend until the age of 74 and an increase from there. In contrast, and again using LOWESS as a reference, for females with severe disabilities we observed an age distribution characterized by a peak at around the age of 55 and a trough at the age of 74. Furthermore, the observed loneliness scores reported by females with severe disabilities increased at a stronger rate from this age as compared to those found for their male counterparts.

Turning to the econometric analysis, firstly Appendix A Table A1 shows the mean values and standard deviation of the explanatory variables used to estimate our loneliness regression by gender (males and females) and disability status (without disabilities, with a moderate disability, and with a severe disability). In line with other previous studies on disability, in general we found people with disabilities were older, less educated, living in households with a lower number of members and children, with lower household income, and a “relational time index” as compared to people without disabilities. These differences were even stronger when we compared people with severe disabilities to their non-disabled counterparts and those with moderate disabilities. Table 1 includes the results obtained after our loneliness model estimations, which are broken down by gender and including individual disability status as a regressor, among other factors. This table also shows the mean values and standard deviations for all explanatory variables used in the corresponding regression. Overall, our regression results corroborate the need to take into account the individual’s degree of disability in this type of study. For the male sample, we found that the coefficients on “moderate disability” and “severe disability” were positive and statistically significant at the 5% level as compared to the reference category (i.e., people without disabilities). In addition, the magnitude of the coefficient for “severe disability” was greater than that found for “moderate disability” (0.237 versus 0.150 points). For the female sample, we obtained the same outcome, but the magnitude of the coefficient for “severe disability” was double that of the magnitude of the coefficient for “moderate disability” (0.343 versus 0.174 points). These results are consistent with other previous empirical studies on loneliness [38,40,47,70]. Looking at the mean values, we found slightly lower percentages for females with moderate and severe disabilities compared to those found for their male counterparts.

To better fit the loneliness distribution by age shown in Figure 2 (with peaks and dips), we included in our model a functional form for age based on a cubic polynomial. For males and females, we found positive and significant coefficients for “age” and “age3/1000)”, and a negative and significant coefficient for “age2”. Namely, we found higher loneliness scores during adolescence or early adulthood, followed by a decrease across middle adulthood, and once again an increase at older ages, consistent with other existing studies [23]. We also found that those individuals with higher levels of education and household income, having a German nationality, existence of children living in the same household, and having a full-time or part-time job were more likely to report lower loneliness scores as compared to the reference person. In contrast, being single (in particular among males) or living without the partner within the same household contributed to increasing loneliness. Similar to other previous studies [39,71,72,73,74], we found that the higher the “relational time index” (i.e., the frequency of contacts with family members, friends, and neighbors; use of online social networks; frequency of participation in volunteering, sports, cultural activities, and religious events), the lower the loneliness score is. In addition, we found that the effect of this variable on loneliness is stronger for females as compared to that found for males (−0.117 versus −0.059 points).

Finally, Table 2 shows the econometric results after re-estimating our loneliness model for four different age groups: 16–29 years (young people), 30–49 (lower-middle-aged people), 50–65 (higher-middle-aged people), and 65+ (older people). Although these specific age groups may seem somewhat arbitrary, Luhmann and Hawkley [23] pointed out that this classification approach allows consideration of development stages and may provide comparable results to those found in previous studies [26,53,54]. In our case, we additionally divided the second age group (30 to 65 years) defined by Luhmann and Hawkley [23] into two subgroups (30–49 and 50–65 years) in order to reflect the age distributions shown in Figure 2 in a better way (and similar to Nicolaisen and Thorsen [50]), especially for those individuals with severe disabilities. Table 2 shows the strength of the relationships between the variable degree of disability (and others) and the sense of loneliness for several age groups and gender groups. Namely, the strengths of these relationships amongst these groups were different. For example, for males aged 16–29 the magnitude of the coefficient for “moderate disability” was 0.222 points (and the mean value for the variable “loneliness” equaled 1.208) as compared to the reference category (i.e., without disabilities), which was a relatively greater value than those found for the other age groups (e.g., 0.084 points people aged 66 or older, and a mean value for “loneliness” equaling 0.929). Although this pattern was also found for females with moderate disabilities, the magnitudes of the coefficients were greater (according to a test of equality of coefficients) than those found for males with moderate disabilities for the younger (0.422 versus 0.222 points) and older groups (0.128 versus 0.084 points). These findings are in line with previous general population studies [75].

For people with severe disabilities, we detected differing effects of disability on loneliness by gender for each age group. For males, once again we found that loneliness was more prevalent in younger ages (the coefficient was 0.508 points and was significant at 5% with respect to the reference category) and decreased with age, with males aged 66 or over reporting the lowest loneliness scores (the coefficient was 0.179 points). In contrast, we found opposite results for females with severe disabilities, i.e., age correlated positively and significantly with loneliness. For example, the coefficient for “severe disability” for the oldest group was 0.38 points, whereas for the youngest one it was negative but not significant at conventional levels as compared to the reference category (−0.092 points). Furthermore, in general we found greater coefficients for females than males for “severe disability” in all age groups, except the youngest group (16–29 years).

According to the literature on loneliness, in general these differences by age between males and females can be explained by a set of developmental and socio-cultural mechanisms [26]. For example, young people are more aware of individual differences and may experience higher loneliness scores because they are more susceptible to experiencing instability from social networks, peer exclusion and victimization, physical and personality changes, family tensions, individualism, and unfulfilled expectations [76,77,78,79]. These factors may be even more relevant among adolescents with disabilities due to the fact that they are more likely to experience social exclusion, bullying, discrimination, lower educational and recreational opportunities, stigmatization, and marginalization than their non-disabled counterparts [80,81,82,83]. These factors highlight in the existing literature may partly explain our results found for males with moderate or severe disabilities and females with moderate disabilities located in the youngest age group (16–29 years).

Looking at middle-aged adults, work status, income, divorce, and caregiving responsibilities were mentioned as main predictors of loneliness [23]. In our case, all regressions for each age group shown in Table 2 included employment status and household income (not reported but available upon request), among other covariates. Consistent with Luhmann and Hawkley [23], we found a significant effect of household income on loneliness for all age groups, but this effect was stronger for middle-aged males and females (30–49 and 50–65). Examining employment status, we found a similar outcome, and with a high percentage of middle-aged adults having a full or part time job (e.g., 89.1 and 70.1% of males and females aged 30–49 were employed, respectively). According to Luhmann and Hawkley [23], income and employment status contribute to protecting one against loneliness, with mid-adulthood being a period wherein “making, investing, and saving money are more important life goals than during early or late adulthood (p. 955)”.

Finally, older people are more vulnerable to suffering from losses of their spouse, family, friends, social status, and poorer health conditions [84,85,86,87]. These losses may lead older people to experience episodes of anxiety and fear and be more vulnerable to loneliness [88]. According to our results, this vulnerability to loneliness is especially relevant for females with severe disabilities aged 66 or over. As reported by Dahlberg et al. [89], there are important gender differences in the prevalence and predictors of loneliness at older ages. They found that mobility problems and mobility reductions were strong predictors of loneliness, but only among females. In our case, we can assume that older females with severe disabilities are more likely to suffer from these mobility problems and other health limitations as compared to younger females. For example, Perissinotto et al. [32] found a 78% higher risk of experiencing limited physical ability among lonely adults aged 60 years or over. These mobility problems can become important limitations and obstacles to participating in leisure activities and maintaining social contacts, and thereby reporting higher loneliness scores [39,57,90]. This reduction in leisure and social activities is compensated for by more time devoted to more frequent contacts with healthcare providers [91]. However, in our case this limitation in health outcomes seems to be less painful in terms of loneliness among males with severe disabilities at older ages. In contrast, Dahlberg et al. [89] also found that only for males do the level of social contacts and social contact reduction predict loneliness. In our case, the frequency of social contacts with family members, friends, neighbors, and use of social online network was captured by the variable “relational time index” (with a negative and significant coefficient, as shown in Table 1). In any case, we have to bear in mind that “loneliness is not only a health issue among older adults but also among middle-aged adults … and might be related to their specific life phases and the reduction of social networks as they age (Jessen et al. [9], p. 1328)”.

## 4. Conclusions

In this study, we analyzed the gender and age differences in the levels of loneliness reported by people without and with moderate and severe disabilities in Germany. Using data from the SOEP for the years 2013 and 2017, we used a three-item UCLA Loneliness Scale and estimated OLS models of loneliness separately for males and females, which included a set of explanatory variables measuring socio-economic characteristics at individual and household levels. In addition, we re-estimated our model by breaking down the sample into four different age groups (i.e., 16–29 years, 30–49 years, 50–65 years, 65+ years) to take into account the different development stages of life and the specific effects of disability status on loneliness in each stage. The results showed that overall females were more likely than males to report higher levels of loneliness. Although similar results were also found for people without and with a moderate or severe disability, we obtained greater loneliness differentials by gender as the degree of severity increased. Consistent with the previous literature on loneliness and disability, we found that people with disabilities experienced higher levels of loneliness, in particular if the individual had a severe disability, as compared to people without disabilities. However, we identified significant gender differences in terms of loneliness between males and females with severe disabilities, with trends being much stronger for the latter. As for age, we also found clear and differing patterns by gender and disability status. We found a negative linear relationship between loneliness and age for males with moderate or severe disabilities. Namely, we found the highest levels of loneliness among males with disabilities aged 16–29, especially for those with severe limiting disabilities. A similar result was found for females with moderate disabilities. On the contrary, we found a positive relationship between loneliness and age for females with severe disabilities, i.e., those 66 or over reported the highest loneliness scores as compared to those for younger groups.

From a public policy perspective, it is important to combat and reduce loneliness among people with disabilities at the early developmental stages of life. Our results have shown that adolescents with moderate and severe disabilities (except females with severe disabilities) experience higher levels of loneliness as compared to their non-disabled counterpart. According to Maxey and Beckert [83], adolescents with disabilities have lower levels of social skills connected with their disability, which makes it more difficult to establish and keep meaningful connections with other developing peers. For those adolescents with severe disabilities, the situation is even worse, because social interactions among peers and friendship reciprocation are rare or almost null for this population group [92]. Looking at the middle-aged and older adults, our results have shown a significant gender differential at these ages. For example, females aged 66 or over report higher levels of loneliness than their male counterparts. Widowhood, living alone, chronic illness, disability, and functional limitations among older females have been pointed out by the existing literature as the main predictors of loneliness to explain these results. Overall, we have to take into account that individuals in different age groups may also experience loneliness differently, with different impacts of the factors associated with loneliness [50], and each group may require different public interventions [93]. Once again, we have to increase our knowledge on the differences in loneliness scores by gender in order to design and develop public targeted interventions to reduce and prevent loneliness in each case (and also age group). Accessibility has become a crucial and major issue among people with disabilities, wherein the existence of “enabling” environments is a key element to promote and improve their participation into society. Barriers in schools, work environments, physical and built environments, within institutional and government policies, and services and assistance have been traditionally pointed out by the existing literature, and contribute to reducing social interactions and increasing loneliness among people with disabilities. For instance, there still exist many barriers (i.e., physical, attitudinal, and environmental) that limit full access of people with disabilities to leisure environments [94]. In this vein, Pagan [95] found that the participation in the leisure events “social gatherings” and “cultural events” has the strongest effect on the level of life satisfaction reported by people with disabilities, and concluded that it is necessary to create inclusive leisure environments that increase social acceptance and boost their physical and emotional health.

Apart from taking into consideration the gender differences detected in our study in terms of loneliness, the type and degree of disability (as our results show for moderate and severe disabilities) must also be taken into consideration by policy makers, governments, healthcare providers, and disability organizations to reduce the levels of loneliness reported by people with disabilities. In our case, an important limitation of this study is the lack of information in the SOEP data on the type of disability that an individual has. For example, we might expect higher levels of loneliness for those individuals who have intellectual disabilities (e.g., Down syndrome, developmental delay, and Fragile X syndrome) as compared to those with physical disabilities (hearing loss, speech problems, and vision impairments). The availability of new datasets that include this type of information allows us to measure, compare, and take different actions according to the type of disability that each individual has. As for potential future areas of research, we could explore the effects of disability among adolescents on the levels of loneliness reported by their parents or other persons living in the same household. It would also be of interest to carry out some longitudinal analyses (using two or more waves) of loneliness in order to identity different disability trajectories over time, and their impacts on loneliness. However, one of the main concerns in analyzing the particular situations relating to various disability groups is the size of the available sample use to construct loneliness and disability measures. In datasets for the whole population, the sample sizes for people with disabilities are traditionally low, whereas in those datasets aimed at examining the specific situation of people with disabilities, the sample sizes are greater. However, these datasets are less frequent, and in many cases quite old. Finally, additional studies on participation in leisure activities among people with disabilities would be very useful in order to understand the main determinants of their levels of loneliness by age, type of disability, and intensity of participation.

## Figures and Tables

**Table 1:** Loneliness regressions for German males and females.

	MALES		FEMALES	
	Mean (SD)	Coefficient (SE)	Mean (SD)	Coefficient (SE)
**Disability status:**				
Without disabilities (*reference*)	0.866	-	0.889	-
Moderate disability	0.071	0.150 *** (0.0214)	0.060	0.174 *** (0.023)
Severe disability	0.063	0.237 *** (0.026)	0.051	0.343 *** (0.028)
Age	51.635 (17.13)	0.087 *** (0.009)	50.935 (16.82)	0.055 *** (0.008)
Age2/1000	2.959 (1.80)	−1.605 *** (0.168)	2.877 (1.77)	−1.106 *** (0.165)
Age3/1000	183.071 (156.18)	0.009 *** (0.001)	175.879 (155.18)	0.006 *** (0.001)
Years of education	12.598 (2.82)	−0.006 *** (0.002)	12.382 (2.71)	−0.005 ** (0.002)
**Marital status:**				
Single	0.159	0.181 *** (0.019)	0.129	0.097 *** (0.019)
Living without partner	0.090	0.211 *** (0.021)	0.186	0.172 *** (0.016)
Living with partner (*reference*)	0.751	-	0.685	-
Existence of children in the household	0.541	−0.020 ** (0.009)	0.593	−0.018 * (0.009)
Household size	2.697 (1.33)	0.009 (0.007)	2.644 (1.34)	−0.011 (0.007)
German	0.923	−0.115 *** (0.023)	0.919	−0.096 *** (0.021)
Log (real household income)	5.416 (0.55)	−0.108 *** (0.012)	5.338 (0.55)	−0.114 *** (0.011)
**Employment status:**				
Full-time	0.552	−0.152 *** (0.017)	0.251	−0.147 *** (0.015)
Part-time	0.040	−0.137 *** (0.026)	0.244	−0.135 *** (0.014)
Non-working (*reference*)	0.407	-	0.505	-
Relational Time Index (RTI)	1.493 (0.56)	−0.059 *** (0.010)	1.529 (0.56)	−0.117 *** (0.010)
Regional dummies		Yes		Yes
Year dummies		Yes		Yes
Constant	-	0.491 *** (0.152)	-	1.284 *** (0.143)
Number observations		19,763		22,806
R-squared		0.065		0.068

Note: Individuals aged 16 or over. The standard errors (SE) are robust. * Significant at *p* < 0.1; ** significant at *p* < 0.05; *** significant at *p* < 0.01%. Source: Author’s calculations using the SOEP data for the years 2013 and 2017.

**Table 2:** Loneliness regression values by different age groups for German males and females.

	MALES							
	16–29		30–49		50–65		+65	
	Mean (SD)	Coefficient (SE)	Mean (SD)	Coefficient (SE)	Mean (SD)	Coefficient (SE)	Mean (SD)	Coefficient (SE)
Disability status:								
Without disabilities (*reference*)	0.978	-	0.946	-	0.820	-	0.756	-
Moderate disability	0.010	0.222 ** (0.041)	0.032	0.201 *** (0.060)	0.106	0.164 *** (0.033)	0.112	0.084 *** (0.032)
Severe disability	0.012	0.508 *** (0.146)	0.022	0.216 *** (0.077)	0.074	0.252 *** (0.044)	0.132	0.179 *** (0.035)
Constant	-	9.029 (5.963)	-	1.378 (3.696)	-	26.59 (21.72)	-	16.68 (13.00)
Number observations		2490		6488		6074		4711
R-squared		0.039		0.088		0.089		0.067
	**FEMALES**							
	**16–29**		**30–49**		**50–65**		**+65**	
	**Mean (SD)**	**Coefficient (SE)**	**Mean (SD)**	**Coefficient (SE)**	**Mean (SD)**	**Coefficient (SE)**	**Mean (SD)**	**Coefficient (SE)**
Disability status:								
Without disabilities (*reference*)	0.983	-	0.955	-	0.833	-	0.805	-
Moderate disability	0.008	0.422 ** (0.209)	0.029	0.160 *** (0.056)	0.105	0.186 *** (0.032)	0.078	0.128 *** (0.039)
Severe disability	0.009	−0.092 (0.157)	0.016	0.260 *** (0.078)	0.062	0.334 *** (0.047)	0.117	0.380 *** (0.039)
Constant	-	4.826 (6.504)	-	2.051 (3.419)	-	−9.316 (22.31)	-	27.33 ** (11.50)
Number observations		2631		8331		6960		4884
R-squared		0.038		0.071		0.096		0.083

Note: All regressions include the rest of the explanatory variables shown in Table 2. The standard errors (SE) are robust. ** Significant at *p* < 0.05; *** significant at *p* < 0.01%. Source: Author’s calculations using the SOEP values for the years 2013 and 2017.

## Appendix A

**Table A1:** Mean values and standard deviations (between parentheses) of explanatory variables used in Table 1 by disability status and gender.

	Without Disabilities		Moderate Disability		Severe Disability	
	Males	Females	Males	Females	Males	Females
Age	49.90 (16.99)	49.58 (16.73)	61.21 (12.40)	59.16 (11.88)	64.65 (14.17)	64.89 (14.05)
Age2/1000	2.78 (1.75)	2.74 (1.74)	3.90 (1.46)	3.64 (1.38)	4.38 (1.75)	4.41 (1.75)
Age3/1000	167.9 (150.4)	164.4 (151.1)	256.6 (137.6)	231.5 (128.3)	307.6 (173.9)	310.2 (175.4)
Years of education	12.72 (2.85)	12.48 (2.73)	11.79 (2.38)	11.93 (2.50)	11.80 (2.51)	11.30 (2.32)
**Marital status:**						
Single	0.17	0.13	0.07	0.08	0.11	0.09
Living without partner	0.08	0.17	0.13	0.25	0.16	0.34
Living with partner	0.75	0.69	0.80	0.67	0.73	0.57
Existence children in household	0.59	0.65	0.22	0.20	0.17	0.13
Household size	2.77 (1.36)	2.72 (1.35)	2.25 (0.98)	2.12 (0.99)	2.15 (1.03)	1.91 (0.92)
German	0.92	0.91	0.95	0.97	0.95	0.97
Log (real household income)	5.44 (0.55)	5.35 (0.55)	5.33 (0.49)	5.27 (0.53)	5.25 (0.50)	5.18 (0.53)
**Employment status:**						
Full-time	0.60	0.27	0.32	0.16	0.13	0.06
Part-time	0.04	0.26	0.04	0.16	0.03	0.06
Non-working	0.36	0.47	0.64	0.67	0.85	0.88
Relational Time Index (RTI)	1.52 (0.56)	1.54 (0.55)	1.41 (0.57)	1.49 (0.57)	1.27 (0.57)	1.34 (0.60)
**Region of residence:**						
Schleswig-Holstein	0.05	0.05	0.04	0.05	0.05	0.06
Hamburg	0.02	0.02	0.01	0.01	0.02	0.02
Lower Saxony	0.10	0.10	0.10	0.09	0.10	0.09
Bremen	0.01	0.01	0.00	0.00	0.00	0.01
North-Rhine-Westfalia	0.20	0.20	0.22	0.21	0.22	0.23
Hessen	0.07	0.07	0.08	0.06	0.07	0.07
Rheinland-Pfalz	0.05	0.05	0.05	0.05	0.04	0.04
Baden-Wuerttemberg	0.12	0.12	0.09	0.10	0.11	0.12
Bavaria	0.16	0.16	0.16	0.14	0.14	0.14
Saarland	0.01	0.01	0.01	0.01	0.01	0.01
Berlin	0.04	0.04	0.04	0.06	0.05	0.04
Brandenburg	0.04	0.04	0.03	0.04	0.05	0.06
Mecklenburg-Vorpommern	0.02	0.02	0.03	0.03	0.02	0.03
Saxony	0.07	0.07	0.06	0.06	0.06	0.05
Saxony-Anhalt	0.04	0.04	0.03	0.03	0.03	0.02
Thuringia	0.04	0.04	0.04	0.04	0.05	0.05
**Year of interview:**						
2013	0.41	0.41	0.48	0.44	0.49	0.48
2017	0.59	0.59	0.52	0.56	0.51	0.52
Number of observations	17,110	20,269	1401	1377	1252	1160
